# Standard MRI-based attenuation correction for PET/MRI phantoms: a novel concept using MRI-visible polymer

**DOI:** 10.1186/s40658-021-00364-9

**Published:** 2021-02-18

**Authors:** Ivo Rausch, Alejandra Valladares, Lalith Kumar Shiyam Sundar, Thomas Beyer, Marcus Hacker, Martin Meyerspeer, Ewald Unger

**Affiliations:** 1 QIMP Team, Center for Medical Physics and Biomedical Engineering, Medical University of Vienna, Waehringer Guertel 18-20/4L, 1090 Vienna, Austria; 2 Division of Nuclear Medicine, Department of Biomedical Imaging and Image-guided Therapy, Medical University of Vienna, Vienna, Austria; 3 High-Field MR Center, Center for Medical Physics and Biomedical Engineering, Medical University of Vienna, Vienna, Austria

**Keywords:** PET/MRI, Phantom attenuation correction, MR visible polymer

## Abstract

**Background:**

PET/MRI phantom studies are challenged by the need of phantom-specific attenuation templates to account for attenuation properties of the phantom material. We present a PET/MRI phantom built from MRI-visible material for which attenuation correction (AC) can be performed using the standard MRI-based AC.

**Methods:**

A water-fillable phantom was 3D-printed with a commercially available MRI-visible polymer. The phantom had a cylindrical shape and the fillable compartment consisted of a homogeneous region and a region containing solid rods of different diameters. The phantom was filled with a solution of water and [18F]FDG. A 30 min PET/MRI acquisition including the standard Dixon-based MR-AC method was performed. In addition, a CT scan of the phantom was acquired on a PET/CT system.

From the Dixon in-phase, opposed-phase and fat images, a phantom-specific AC map (Phantom MR-AC) was produced by separating the phantom material from the water compartment using a thresholding-based method and assigning fixed attenuation coefficients to the individual compartments. The PET data was reconstructed using the Phantom MR-AC, the original Dixon MR-AC, and an MR-AC just containing the water compartment (NoWall-AC) to estimate the error of ignoring the phantom walls. CT-based AC was employed as the reference standard. Average %-differences in measured activity between the CT corrected PET and the PET corrected with the other AC methods were calculated.

**Results:**

The phantom housing and the liquid compartment were both visible and distinguishable from each other in the Dixon images and allowed the segmentation of a phantom-specific MR-based AC. Compared to the CT-AC PET, average differences in measured activity in the whole water compartment in the phantom of −0.3%, 9.4%, and −24.1% were found for Dixon phantom MR-AC, MR-AC, and NoWall-AC based PET, respectively. Average differences near the phantom wall in the homogeneous region were −0.3%, 6.6%, and −34.3%, respectively. Around the rods, activity differed from the CT-AC PET by 0.7%, 8.9%, and −45.5%, respectively.

**Conclusion:**

The presented phantom material is visible using standard MR sequences, and thus, supports the use of standard, phantom-independent MR measurements for MR-AC in PET/MRI phantom studies.

## Background

In the last decade, combined positron emission tomography/magnetic resonance imaging (PET/MRI) has been established in research and clinical practice [1]. For this, several technical and methodological challenges had to be overcome [2–4] the most prominent being the use of MR images for the purpose of attenuation correction (AC) of the PET emission data [5].

MRI is based on the measurement of electromagnetic signals arising from the precession of the bulk magnetization of nuclear magnetic moments in an outer magnetic field after excitation by a radiofrequency pulse. In contrast to CT, the measured signal is not related to the attenuation properties of the investigated material for X-ray and annihilation radiation. Therefore, simple scaling approaches for AC, as used in PET/CT, cannot be applied and new AC concepts had to be developed. Today, several MRI-based AC methods are available, which work sufficiently well for assessing the attenuation properties of a human subject in most standard clinical PET/MRI investigations [6–9], although their accuracy in specific organs such as lung are still a matter of debate [10, 11]. Some attempts have been made to be able to use the standard MR-AC methods also for phantom studies. For example, the use of biological materials to produce realistic phantoms has been proposed [12] and combinations of water saturated plaster, silicone, and agarose gel have been suggested to mimic bone, adipose, and brain tissue, respectively [13]. However, MRI-based AC for hardware components and phantom studies is in general not sufficiently addressed.

Most solid materials, including those used for MRI coils, patient positioning devices or quality control phantoms, are not detectable by standard MRI [14, 15]. Therefore, these materials cannot be taken into account in standard MRI-based AC approaches and their additional attenuation is not corrected for. The consequence thereof is a spatially variant underestimation of the true activity within investigated objects [16–21].

To overcome this issue, several techniques have been investigated. The most commonly employed one is the use of CT- or transmission measurement-based attenuation templates [22]. Here, an attenuation map is generated from a CT or transmission scan of the hardware component or phantom and incorporated into the AC during image reconstruction. This approach has been shown to work well for hardware parts of which the exact position in the scanner FOV is known, such as fixed MRI coils or radiation therapy table tops and for phantom studies if proper phantom positioning aids are used [16, 21]. However, the generation and implementation of such templates are labor-intense and, for phantom acquisitions, additional co-registration steps may be required [16].

For flexible components and components for which the exact position is not known, template-based approaches including MRI-visible markers on the hardware components and non-rigid co-registration have been proposed [23]. Furthermore, PET data-based methods using reconstruction algorithms able to compute the attenuation and activity distribution simultaneously have been investigated [24]. However, these methods are tailored to specific applications, such as radiotherapy planning or the correction for MRI-compatible headphones, and none of these methods has demonstrated general applicability.

In view of the challenges associated with the current approaches for AC of non-stationary hardware parts, and specifically phantoms, alternative approaches are desirable. One option is to visualize the hardware or phantom material with MRI and use simple segmentation-based methods for deriving the correct attenuation map. This would either require the use of ultra-short echo time (UTE) or zero echo time (ZTE) sequences, which are able to visualize solid materials with short relaxation times [25, 26]. Another approach is the use of a suitable solid material visible in standard MRI. One example of such a material was recently published by Mitsouras et al. [27] who used a commercially available 3D printable polymer (Object High Temperature RGD525, Stratasys) to produce an MRI-visible spine phantom. The phantom material produced an MRI signal in gradient echo (GRE) MRI with an intensity of about one third of the intensity found with saline solution, with relaxation times found to be T1 = 194 ms and T2 = 32 ms. This MRI phantom material was further validated by Rai et al. [28] in a series of more complex phantom measurements. The RGD525 has similar material properties as a common standard phantom material for PET phantoms, polymethylmethacrylat (PMMA), also known as acrylic glass [29]. It has a density of 1.17-1.18 g/cm3, a modulus of elasticity of 3.1-3.5 GPa and a water absorption of 1.2-1.4%. Therefore, it seems suitable as building material for fillable PET phantoms [30]. Here, we investigate if AC for PET/MRI phantoms can be performed with standard MR-AC approaches when using an MRI visible polymer as phantom material. Therefore, we built a cylindrical phantom using the MRI-visible, 3D printable polymer RGD525 and evaluated its usability in combination with MRI-based AC methods in a standard PET/MRI system.

## Material and methods

### Phantom

**Fig. 1:**
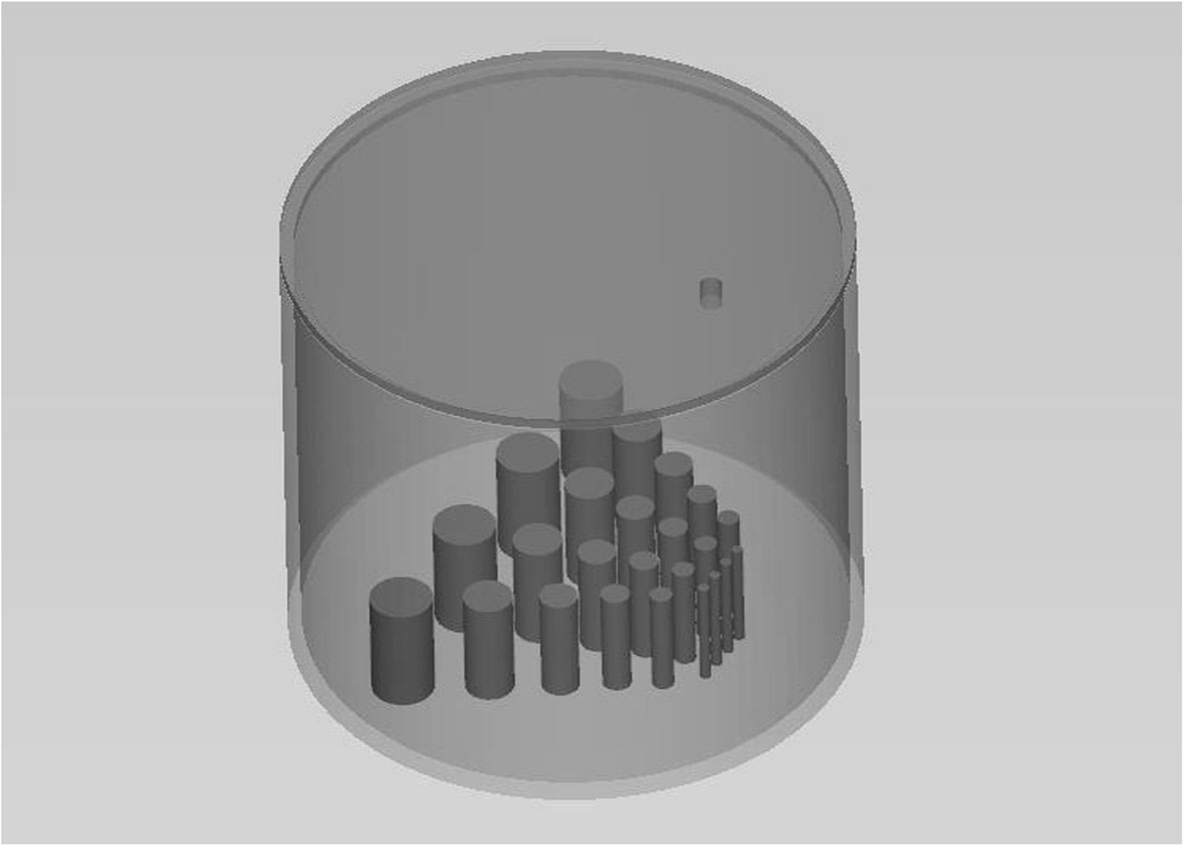
3D mode of the phantom as constructed in CAD. The upper section provides a homogeneous area. The lower sections contain an arrangement of rods with different diameters as used in resolution phantoms. The whole phantom including the rods is made from the same material

The phantom consists of a cylinder with an outer length of 19 cm, an outer diameter of 25.2 cm and a wall thickness of the cylinder shell of 6 mm. The cylinder was closed on the top and bottom with a 10 mm and a 5 mm lid, respectively. This results in a cylindrical inner compartment of 24 cm diameter and 18.5 cm length, which can be filled with liquids (e.g., aqueous solutions containing radioactive isotopes). The top lid contained a sealable drilling to allow filling the phantom. At the bottom lid, arrangements of rods of 5 cm length and with five different diameters of 5 mm, 10 mm, 13 mm, 17 mm, 22 mm, and 28 mm were attached. The chosen arrangement was similar to an MRI resolution phantom [31], but with the rod diameters adjusted to the resolution of a whole-body PET system. Therefore, the phantom presents a homogeneous region in the upper part and a heterogeneous region near the bottom (Fig. 1).

The whole phantom was constructed using an open source computer-aided design (CAD) program (FreeCAD, Version 0.18, www.freecadweb.org). The design consisting of three parts, the cylinder, the top lid, and the test array was exported as an STL-file as an input for the 3D printer. For 3D printing, we used an MJM-printer Connex3 Objet 500 (Stratasys, Rechovot, Israel), which provides a resolution of 600 dpi for the *x*/*y*-coordinates and 800 dpi for the *z*-coordinate. The phantom was printed from high-temperature UV-curing material RGD525 in its pure quality. The printing of the model was done orthogonal to the axis of the cylinder with a layer thickness of 30 μm. Based on the dimension of the phantom, printing time was 79 h whereby 1.9 kg of the building material was needed.

### PET/MRI measurements

The measurements were performed on a clinical PET/MR system (Biograph mMR, Siemens Healthineers, Erlangen, Germany). The phantom was filled with a solution of water and [18F]fluoro-2-deoxy-d-Glucose ([18F]FDG) with an activity concentration of 10 kBq/ml. Sodium chloride (0.9%) and 5 ml MR contrast agent (Dotarem, Guerbet, France) were added to avoid MR artifacts [32]. The phantom was placed on the PET/MRI patient bed and positioned in the center of the field-of-view with the main axis of the phantom parallel to the main axis of the PET/MRI system. A 30-min PET acquisition was performed including the standard Dixon-based MR-AC method as implemented in software version VB11P (Table 1). MR imaging for the MR-AC was performed using the integrated body coil. No other MR coils were used during acquisition to limit additional attenuation caused by the hardware. The output of the Dixon MR-AC was an in-phase, opposed-phase, fat and water image, and ultimately the segmentation-based Dixon MR-AC map.

**Table 1:** Parameters of the standard Dixon MR-AC method as implemented for whole-body examinations

Parameter	Value
Echo time (TE1/TE2)	1.23 ms/2.46 ms
Repetition time (TR)	3.96 ms
Flip angle	9°
Slice thickness/slice gap	3.1 mm/0 mm
Pixel spacing	2.6 × 2.6 mm^2^
Matrix size/number of slices	192 ×120/128
Phase encoding direction	AP

After the PET/MRI acquisition, a CT scan of the phantom was acquired on a Biograph TPTV PET/CT system (Siemens Healthineers, USA). CT scan parameters were tube voltage = 120 kV, tube current time product = 160 mAs, pitch = 0.8, slice thickness = 5 mm, in-plane pixel size 1.4×1.4 mm^2^ using a 512×512 matrix.

### Attenuation correction and image reconstruction

Four different attenuation maps were created, whereby the standard, CT-AC map served as the reference standard. The standard Dixon-based MR-AC (“Standard MR-AC”) was generated using the method as implemented for whole-body AC in the PET/MRI system. The same data that were the base of the standard MR-AC approach were used to create a phantom-specific MR-AC (“phantom MR-AC”). Finally, an AC map only containing the water compartment (“NoWall MR-AC”) was created to imitate a typical phantom (as provided by the vendors), with a wall material that is not visible in standard MR-AC.

Both, the phantom- and the NoWall MR-AC were created using a simple thresholding approach as follows. First, the total volume of the phantom was estimated from the “opposed-phase” MRI. A mask was produced by thresholding all pixels with values > 100 in the opposed phase MRI followed by a morphological closing operation to account for pixels with values < 100 within the phantom. The final mask representing the total phantom was defined by selecting the biggest connected compartment within the threshold mask followed by isotropic 1-voxel erosion. These steps were necessary to exclude artificial pixels at the edge of the MRI FOV and to account for an overestimation of the phantom extent caused by the low threshold, respectively.

Second, the water compartment was determined. The Dixon fat images were filtered with a Gaussian (sigma = 0.5 pixel) and all values < 10 where set to zero. Then the fat image was subtracted four times from the opposed phase image to enhance the contrast between the water and the phantom material compartment. This image was then used to produce a mask of the water compartment by thresholding all pixel values > 400 followed by a morphological closing operation to account for individual pixels with values < 400 within the water compartment. The phantom material compartment was obtained by subtracting the mask of the water compartment from the mask of the total phantom. The final phantom MR-AC was created by assigning a linear attenuation coefficient for 511 keV photons of 0.096 cm^−1^ and 0.1037 cm^−1^ to the water and phantom material, respectively. These values were obtained from the median of the linear attenuation coefficients from standard CT-AC. The NoWall MR-AC was created similarly with the only difference being that the phantom material was set to zero attenuation.

The CT-AC was created by registering the CT images to the in-phase MRI and converting the CT units into linear attenuation coefficients using a bilinear scaling approach [33].

PET data reconstruction was performed with the four AC maps. Reconstructions were done using vendor-based software (e7tools, Siemens Healthineers, USA) with an ordinary-Poisson ordered-subsets expectation maximization (op-OSEM) algorithm with 3 iterations and 24 subsets. Image matrix size was 344 × 344 × 127, and a 5-mm FWHM Gaussian post-reconstruction filtering was applied.

### Data evaluation

AC maps and PET images were visually compared including an evaluation if the rod structure can be used to visually assess the PET resolution with the different AC methods similar as done in MRI or for single photon computed tomography (SPECT) using the Jaszczak Phantom [31].

Voxel-based %-difference images between the different MR-AC maps and the CT-AC were calculated. Similarly, relative %-difference images between the MR-AC-corrected PET images and the CT-AC PET were calculated.

To assess the impact of the different AC methods on quantitative PET readings, the average difference in activity concentration was calculated in the following regions of the phantom: (a) in the whole water compartment (by thresholding the CT-AC), (b) in a slice of the homogeneous region from an ROI including the whole water compartment in that slice, and additionally, in a 5 pixel wide ROI at the border of the phantom walls. (c) In a slice of the heterogeneous region from an ROI including the whole water compartment in that slice, and additionally, in a 5 pixel wide ROI around all rods and the phantom housing.

## Results

**Fig. 2:**
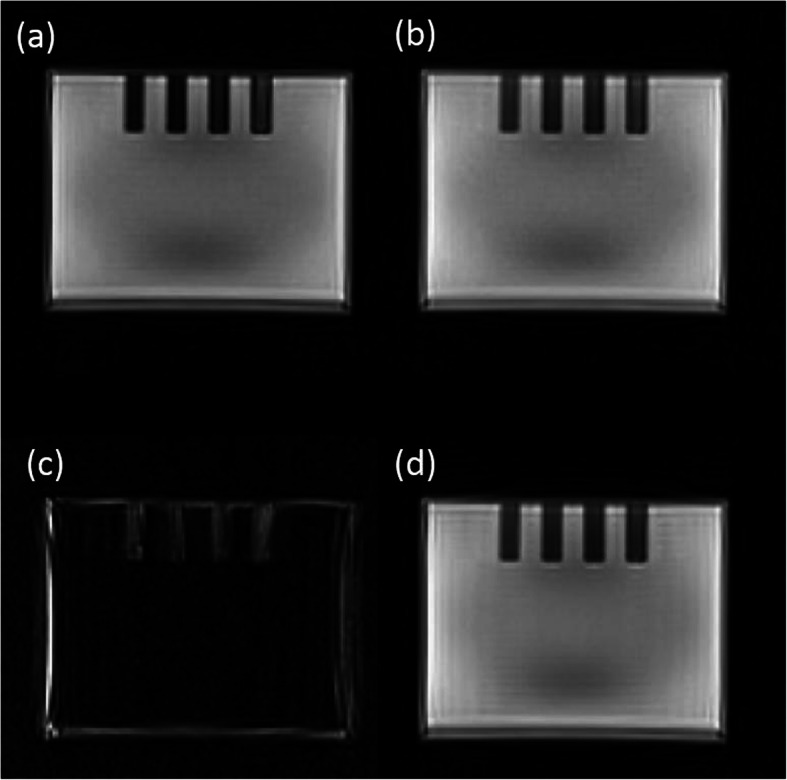
Dixon MR images from the standard Dixon-based AC approach in the Biograph mMR PET/MR system. **a** in-phase image, **b** opposed-phase image, **c** “fat” image, **d** water image

**Fig. 3:**
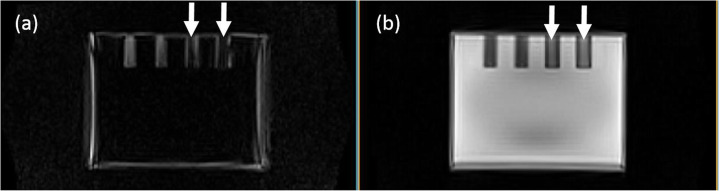
Tissue swap-like artifacts (white arrows) in the “fat” (**a**) and “water” (**b**) image

The phantom housing and the liquid compartment were both visible and visually distinguishable from each other in the Dixon in-phase and opposed-phase images (Fig. 2). However, the phantom material signal was only partly separated from the liquid compartment using the Dixon water-fat separation, as the Dixon water image still contained signal from the phantom housing (Fig. 2). Furthermore, the water compartment showed a global reduction of signal intensity toward the center of the compartment. In the central parts of the rods in the heterogeneous region, the Dixon water and fat images showed signal voids in the fat image with corresponding higher signal intensities in the water image (Fig. 3).

The standard MR-AC included the phantom material, but did not separate the liquid compartment from the phantom material. The entire phantom was treated as soft tissue and a corresponding linear attenuation coefficient of 0.1 cm^−1^ was assigned. In general, the standard MR-AC overestimated the phantom extent in the order of one pixel (2 mm).

**Fig. 4:**
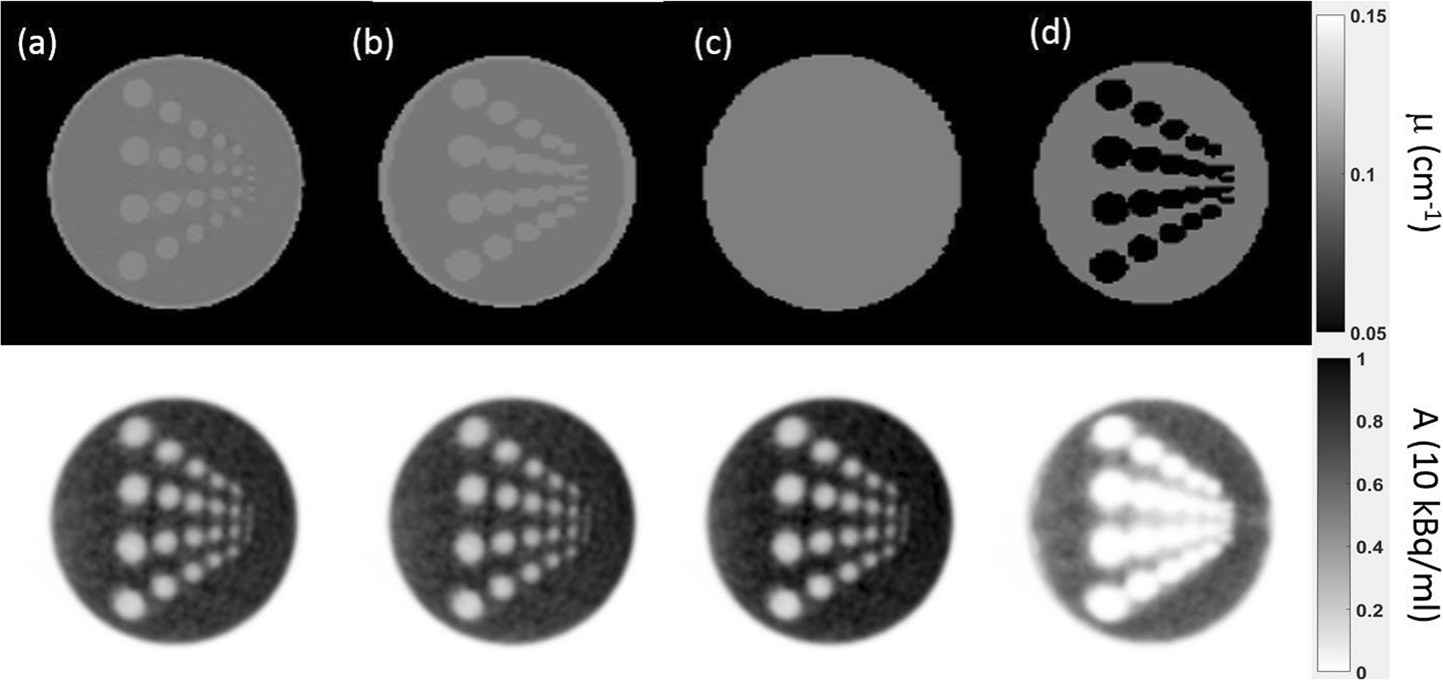
AC (top) and corresponding PET (bottom) images. **a** CT-AC, **b** phantom MR-AC, **c** standard MR-AC, **d** NoWall MR-AC

**Fig. 5:**
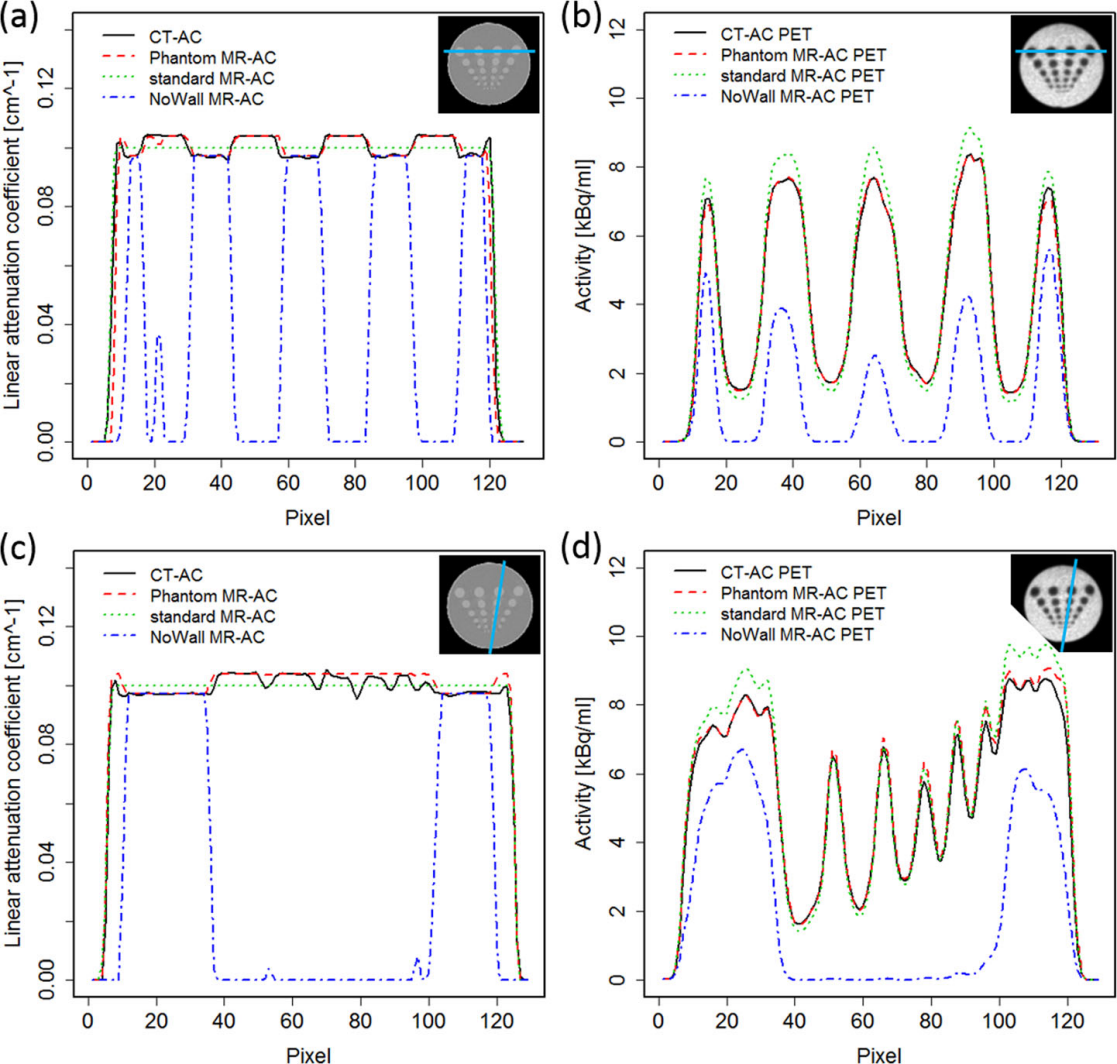
Line profiles through the AC maps and corresponding PET images. Profile through the 28 mm rods in direction parallel to the Dixon MRI slice orientation for (**a**) the AC maps and (**b**) the corresponding PET images. Profile through the different diameter rods in direction nearly perpendicular to the Dixon MRI slice orientation for (**c**) the AC maps and (**d**) the corresponding PET images

**Fig. 6:**
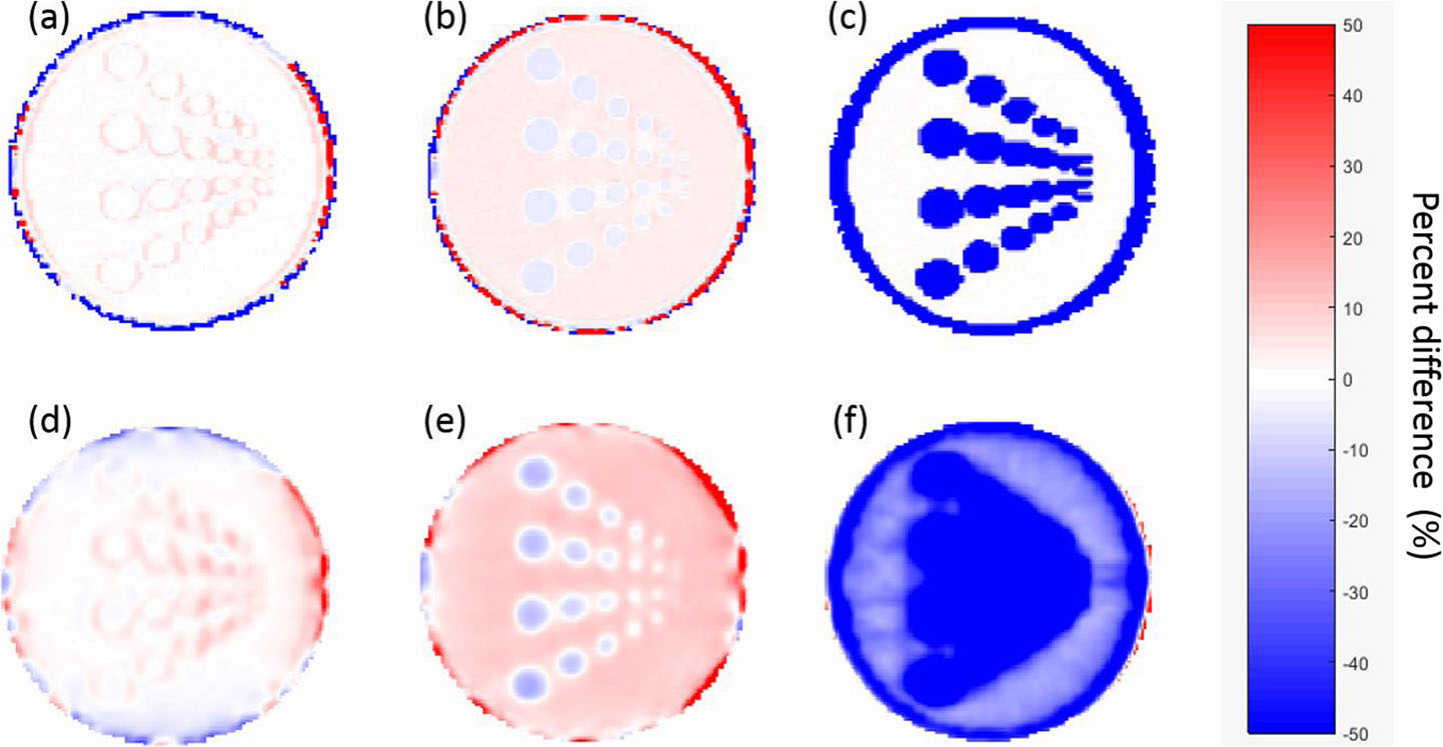
Percent difference images. Top row depicts the percent difference between the MR-AC maps and the CT-AC for (**a**) phantom MR-AC, **b** standard MR-AC, and **c** NoWall MR-AC. The bottom row depicts the percent difference images between the PET corrected with the different MR-AC approaches and the CT-AC PET: **d** phantom MR-AC PET, **e** standard MR-AC PET, and **f** NoWall MR-AC PET

The segmentation approach used for the phantom MR-AC did separate the phantom material from the water compartment. However, an overestimation of the extent of the phantom material within the phantom of in general about one pixel was seen (Fig. 4). This overestimation was not uniform and more pronounced in directions perpendicular to the MRI slices. This resulted in an artificial conjunction of some of the rods in this direction (Fig. 5a and c). Figure 6 shows %-difference images for the different MR-AC maps.

PET images reconstructed with the standard and phantom MR-AC were visually comparable to the CT-based reconstructions. The rod structures were visible and distinguishable from each other down to a rod diameter of 10 mm in the PET images except the ones corrected using the NoWall MR-AC (Fig. 4). The NoWall MR-AC-based PET reconstructions presented visually with substantially underestimated activity in the heterogeneous region of the phantom (Fig. 4).

Compared to the CT-AC PET, average differences (±SD) in measured activity in the entire water compartment of −0.3 (±2.1) %, 9.4 (±1.9) %, and −24.1 (±12.9) % were found for phantom MR-AC, standard MR-AC, and NoWall MR-AC-based PET, respectively. The average difference of the measured activity in a slice of the homogeneous region was −0.5 (±2.3) %, 9.1 (±2.0) %, −23.1 (±11.7) %, respectively. The average difference of the measured activity in a slice of the heterogeneous region was 0.5 (±2.2) %, 9.1 (±1.7) %, and −37.6 (±21.4) %, respectively.

Average differences near the phantom wall in the homogeneous region were −0.3 (±4.3) %, 6.6 (±1.9) %, and −34.3 (±16.8) %, respectively. Around the rods, activity differed from the CT-AC PET by 0.7 (±2.6) %, 8.9 (±2.2) %, and −45.5 (±22.4) %, respectively. The artificial conjunction of the rods in vertical direction in the phantom MR-AC did only minimally influence the PET quantification between the rods (Fig. 5b and d). Difference images between the MR-AC PET reconstructions and the CT-based PET are shown in Fig. 6.

## Discussion

We show that the phantom housing can be visualized in MRI using standard sequences and that it is recognized and incorporated in the standard MR-AC approach. Using a dedicated segmentation approach for the phantom MR-AC, average bias in activity measurements are below 1% compared to CT-based AC. Furthermore, PET data reconstructed using the standard as well as the phantom MR-AC method resulted in PET images visually comparable to CT-AC-based PET.

Using the standard MR-AC approach for whole-body imaging, the whole phantom was recognized as soft tissue. The reason is that the Dixon water-fat separation was not able to fully separate the phantom material from the water content (Fig. 2). The Dixon water-fat separation relies on intra-voxel dephasing due to the different phase evolution of lipid (with its most abundant component, the methylene protons resonating at 1.3 ppm) and tissue water signals (resonating at 4.65 ppm) [34]. Yet, to the best of our knowledge, the phantom material does not contain a pure water component, and, thus, a Dixon contrast for the material is not expected. Only for the voxels at the phantom-water border, a partial volume effect can lead to intra-voxel dephasing, which can be exploited to deliver Dixon “fat-water” contrast (Fig. 2). Of note, this effect was used in the phantom MR-AC approach to enhance the borders between the phantom material and the water compartment for a better performance of the segmentation by subtracting the Dixon “fat” image from the opposed-phase images.

It would be possible to further enhance these partial volume effects by proper adjustment of the Dixon sequence. Rai et al. have identified the main peak in the ^1^H spectrum for the used material at 3.5 ppm [28]. The frequency difference between water (at room temperature the chemical shift is approx. 4.8 ppm) and the polymer’s main peak resonates at ca. 1.3 ppm. Therefore, the standard TEs used for the Dixon water-fat separation (1.21 ms and 2.42 ms) are inappropriate to generate opposed-phase and in-phase images with this material, and the correct TEs for this setting would be 3.39 ms and 6.78 ms. With further optimizations, using multi-point measurements with additional TEs, also the less abundant component at 1.0 ppm could be captured, but at such short echo times its contribution can be neglected. However, such adjustments are not possible in the standard MR-AC implementation, and therefore, this was not done in this study.

Using an adjusted segmentation approach enabled the separation of the phantom material from the water compartment. Here, the main challenges were global signal variations and tissue-swap-like artifacts in the Dixon MRI images. Signal variations over the field of view are expected due to inhomogeneous RF excitation and reception, which could in principle be mitigated, at least partly, by employing phased array RF coils [35] and *B*_1_^+^ shimming [36] or with adiabatic RF pulses [37]. However, the use of additional RF coils may induce again at least a local bias in PET quantification if their attenuation is not accounted for [20]. With the dimensions of the phantom (25 cm diameter) being on the order of the RF wavelength in tissue or water at 3 T, local signal amplification or attenuation due to wave effects are, however, unavoidable. The signal is expected to be significantly more spatially homogeneous with substantially smaller phantoms or at lower magnetic field strengths.

In general, tissue swap artifacts are well known in Dixon-based water-fat separation [38]. They are caused by local magnetic field inhomogeneities and have been frequently observed in MR-AC [39, 40]. Their prevalence may be reduced by using advanced water-fat separation algorithms [41] but will not be completely avoidable. We hypothesize that the tissue-swap-like artifact observed (Fig. 3) has a similar cause like the well-known tissue swap artifacts. However, the tissue-swap-like occurred only within the phantom material. With the used segmentation algorithm based on thresholding the whole phantom and the water compartment, these tissue swaps did not translate into the phantom MR-AC.

In general, both MR-AC methods (standard and phantom) were challenged by reproducing the exact geometry of the phantom as seen on CT images. The standard MR-AC overestimated the extent of the phantom by approximately one pixel (2 mm). The phantom MR-AC did overestimate the extent of the 3D-printed phantom material within the object which was more pronounced in direction perpendicular to the original MRI slices (Figs. 4 and 5). This is expected to result from partial volume effects in the original Dixon images as the slice thickness is greater than the in-plane pixel size (Table 1). In addition to the partial volume effects, the use of the Dixon “fat” images to improve the contrast at the borders between the material and the water compartment and the selected threshold for the object segmentation may also contribute to the general overestimation of the phantom material extent. Such inaccuracies in reproducing the correct outline of an object have already been observed in MR-AC of the head [42] and may be reduced by a refinement of the segmentation procedure and the adjustment of the voxel size in the Dixon MRI sequence used for MR-based AC generation.

The local inaccuracies in reproducing the phantom housing material extent are also assumed to cause the local over- and under-estimations near the phantom walls in the phantom AC-based PET (Fig. 5). A refinement of the segmentation procedure is expected to further reduce these local biases. However, already in the current implementation using a rather simple segmentation approach, the general quantitative bias of the PET was < 1%. Such a bias is negligible for most applications, such as cross calibration or NEMA image quality measurements. Thus, the presented concept can be readily used in its current form for a broad range of phantom experiments without the need of CT-based attenuation templates.

### Limitations

The main limitation of the presented approach is that the phantom MR-AC segmentation procedure has been developed and tested only for one phantom. Specifically, the selection of the used thresholds was based on an empirical inspection of the voxel value distributions. To generalize the segmentation procedure for the use with a broad range of phantoms, the segmentation and threshold selection would require an optimization using a variety of phantoms with different shapes, wall thicknesses, and sizes. Furthermore, the applied morphological closing algorithm may cause artifacts when using phantoms containing air-filled cavities. However, with an optimized MR-AC sequence with improved homogeneity and improved separation of the water and phantom housing compartment, the morphological closing may not be needed.

## Conclusion

The presented MRI-visible polymer phantom housing is detectable using standard Dixon-based MR-AC sequences, and thus, can be incorporated in a routine MR-AC algorithms. When combining standard Dixon-based imaging with a dedicated thresholding approach, phantom-specific MR-AC maps were created that yielded a residual bias of the AC-PET of 1% or less.

## Data Availability

The datasets used and/or analyzed during the current study are available from the corresponding author on reasonable request.
